# Association of the MC4R gene with growth traits and meat quality in Colombian hair sheep

**DOI:** 10.5455/javar.2023.j698

**Published:** 2023-09-24

**Authors:** Darwin Yovanny Hernández-Herrera, Diego Fernando Carrillo-González, Juan Carlos Rincón-Flórez

**Affiliations:** 1 Departamento de Ciencia Animal, Facultad de Ciencias Agropecuarias, Grupo de Investigación en Recursos Zoogenéticos, Universidad Nacional de Colombia Sede Palmira, Palmira, Colombia; 2 Departamento de Zootecnia, Facultad de Ciencias Agropecuarias, Grupo de Investigación Mejoramiento Genético Animal, Universidad de Sucre, Sincelejo, Colombia

**Keywords:** Colombian creole sheep, cook-loss, longissimus thoracis et lumborum muscle, meat texture, melanocortin receptor 4, preweaning growth, postweaning growth

## Abstract

**Objective:**

The objective of this study was to associate the 1016G > A variant of the melanocortin-4 receptor gene with lamb’s weight, growth, and meat quality in the Colombian hair sheep breed.

**Materials and Methods:**

A total of 168 lambs, weights were measured at birth weight (BW), at weaning adjusted weaning weight (AWW), at 6 months [adjusted weight (AW180)], at slaughter adjusted slaughter weight (ASW), daily weight gain preweaning daily gain (preWDG), and postweaning daily gain (postWDG) weaning, and after slaughter, pH, texture, and cook-loss (CL) in the *longissimus thoracis et lumborum* (LTL) muscle according to the American Meat Science Association methodology. The 1016G > A genotypes were obtained by sequencing. Genotypic and allele frequencies, heterozygosities, and Hardy–Weinberg equilibrium (HWE) were estimated. Using a generalized linear model, the genotype and the allelic substitution effect were associated with the evaluated traits.

**Results:**

The heterozygous genotype (0.48) and G allele (0.61) were the most frequent. Heterozygosities were similar (0.47), indicating HWE. The genotype affected the BW (*p* < 0.05), with a higher value for the GG genotype (2.69 kg). AWW (12.75 kg), AW180 (19.67 kg), and ASW (31.21 kg) weights and daily weight gain (preWDG = 115.41 gm; postWDG = 96.16 gm) were not associated. Average pH, Warner-Bratzler shear force, and CL were 5.75 units, 49.46 N, and 32.02%, with no genotype effect. The G > A substitution only affected BW at −388 gm (*p* < 0.05).

**Conclusion:**

The 1016G > A variant is polymorphic and affects the BW but not other growth traits or the meat quality of the LTL muscle.

## Introduction

The Colombian hair sheep (OPC) is the base breed of the productive systems in Colombia. Their adaptative capacity allows them to be found throughout the country. However, the different levels of technological inclusion in the production systems are associated with very heterogeneous productivity regarding growth performance and product quality [1].

Based on the above, to standardize the production process, efforts have been made to slaughter animals weighing more than 30 kg and under 1 year of age. In this sense, various investigations have characterized the growth of OPC in various production conditions [2–5]. However, studies on carcass quality [6,7] and meat [8] are few. The above may be because the concept of quality is very broad, but it can be summarized as the set of attributes that satisfy the needs of the final consumer; these attributes can be organoleptic, nutritional, safe, commercial, technological, and image. The last one includes the cultural, ethical, and environmental dimensions of the production [9].

Advances in genomic association studies have made it possible to identify candidate genes with a greater effect on growth traits and carcass and meat quality [10]. One of these candidate genes is the melanocortin-4 receptor (MC4R, GeneID: 100147707) [11], which has not been studied in the OPC [12]. MC4R is one of the five currently identified melanocortin receptors that belong to the G-protein receptor family and encodes a seven-domain transmembrane protein [13]. This gene is expressed in the hypothalamic paraventricular nucleus. It is an important component in the leptin-melanocortin signaling pathway. MC4R is activated by neuropeptides α- and β-MSH derived from proopiomelanocortin, whereas, in the arcuate nucleus, it can be blocked by Agouti-related peptide (AgRP) produced by AgRP/neuropeptide Y neurons, which regulate their function by the action of leptin and ghrelin that come from adipose tissue or the intestine [11].

According to some authors, a polymorphism located in exon seven of this gene increases food consumption by around 10%, with a consequent increase in weight gain per day (6%–10%), reaching slaughter weight faster (68%), and affecting carcass conformation traits [13,14]. Despite the above, the effect of this genetic variation on traits related to meat quality has not been studied. Therefore, this work aimed to associate the 1016G > A variant of the MC4R gene with the traits of growth and meat quality in the *longissimus thoracis et lumborum* muscle (LTL) in Colombian hair sheep (OPC).

## Materials and Methods

### Ethical approval

The experimental procedures used in this research were approved by the Committee of Animal Care and Welfare of the Universidad de Sucre according to approval letter number 11-2020.

### Study location

This work was carried out with lambs from the Córdoba (8°22�0�� N, 75°42�0�� W) and Sucre (9°2�0�� N, 75°9�0�� W) departments with an elevation of 63 and 31 mamsl, respectively, a temperature range of 26°C–28°C, and a relative humidity of 75%. Both are classified as tropical dry forests [15] within the Caribbean region of Colombia. In addition, by the year 2022, this region will maintain 80.3% of the country‘s sheep inventory [16].

### Animal management

168 Colombian-hair sheep (OPC) lambs were used, fed on *Braquiaria brizanta* and *Bothriochloa pertusa* grazing between 8:00 and 17:00 h with continuous access to mineralized salt (6%) and water ad libitum. In addition, they were offered star grass hay (*Cynodon plectostachyus*, dry matter: 85.7%, crude protein: 3.9%, ash: 15.2%, ethereal extract: 2.1%, NDF: 65.1%, ADF: 36.9%, lignin: 8.9%, Ca: 0.64%, P: 0.07%, and 2.2 Mcal/Kg DM of digestible energy) and corn silage (dry matter: 27.1%, crude protein: 8.5%, ash: 4.9%, ethereal extract: 2.1%, NDF: 53.3%, ADF: 28.2%, lignin: 5.0%, Ca: 0.22%, P: 0.35%, and 3.0 Mcal/Kg DM of digestible energy) by creep feeding in pens during the night. The lambs were vaccinated against *Pasteurella* and *Clostridium* 15 days before weaning, which was performed between 85 and 95 days old, and later, selective deworming was carried out every 30 days, according to the score obtained by the FAMACHA^®^ method.

The traits of birth weight (BW), adjusted weaning weight at 90 days (AWW), 6-month adjusted weight (AW180), and adjusted slaughter weight at 300 days (ASW) were assessed in the lambs. Likewise, preweaning daily gain (preWDG) and postweaning daily gain (postWDG) were estimated.

### DNA extraction and genotyping of the MC4R gene

During the AW180 measurement, 3 ml of peripheral blood was obtained by jugular vein puncture in tubes with anticoagulant (EDTA 7.2 mg). DNA extraction and purification were performed using the commercial QIAamp^®^ DNA Mini Kit, according to the manufacturer’s instructions [17]. DNA quality was assessed qualitatively using 0.8% agarose gels stained with GelRed™ (Biotium) and quantitatively using NanoDrop 2000™ (Thermo Fisher Scientific). All samples were diluted to 10 ng/µl for use in polymerase chain reaction (PCR).

In an Eppendorf^®^ MasterCycler Nexus Gradient thermocycler, using the primers 5�-CCT GCA CCT GAT ATT CTA CAT TCT C-3� and 5�-GTA CAC ATG AGA AGG AGA AGG TC-3�, in reactions with a final volume of 50 μl containing 20 pmol of each primer, 20 ng of DNA, and 1X of the MangoMix™ supermix (Bioline^©^), a 274 base pair fragment was amplified [13]. The amplification protocol consisted of an initial denaturation at 94°C for 3 min, followed by 35 cycles of denaturation at 94°C for 30 sec, annealing at 60°C for 30 sec, extension at 72°C for 45 sec, and a final extension at 72°C for 10 min [13]. Amplified products were visualized on 1.4% agarose gels stained with GelRed™ (Biotium).

The amplified samples were sequenced in both directions in the MACROGEN company. Electropherograms were edited using the Geneious Prime 2022.1 software (https://www.geneious.com), the sequences were compared using BLAST software (http://blast.ncbi.nlm.nih.gov/Blast.cgi), and using the MEGA X software the sequences were aligned [18]. The animals were genotyped according to the 1016G>A polymorphism [13,19].

### Postmortem assessment

The slaughter age was 320 ± 20 days. The animals were fasted for 12 h and then weighed. The weight was adjusted at 300 days (ASW). Slaughtering was carried out in a commercial slaughterhouse located in Cereté, Colombia, by stunning with a captive bolt gun and slaughtering in accordance with Colombian standard operating procedures (Decree 1500 of 2007 of the Ministry of Social Protection). After bleeding, the hot carcasses were refrigerated at 4°C. At 24 h postmortem, the carcasses were divided in half, and on the right side of the carcass, two 2.54 cm thick fillets were taken, sequentially extracted from the LTL muscle between the third and eighth thoracic vertebra [20].

The pH of the meat was determined 24 h postmortem with a puncture pH meter (CRISON GLP22, Alella, Barcelona, Spain) in each of the fillets, puncturing the center of the LTL muscle section with the electrode [21]. Later, the fillets were vacuum packed and kept between 2°C and 4°C for future measurements [22].

The texture of the meat was measured instrumentally through the WBSF [23]. Briefly, 48 h postmortem, the LTL muscle samples were cooked in plastic bags at 75ºC in a water bath until reaching an internal temperature of 70ºC, which was maintained for 20 min [22]. The internal temperature of the samples was monitored using a digital thermometer (DM6801A, Shenzhen Victor Hi-tech Co. Ltd., Shenzhen, China). Once the sample was cold, four cylindrical nuclei of 1.27 cm in diameter were extracted from each one, parallel to the orientation of the muscle fiber. WBSF measurements were performed with the TA-XT2i machine (Godalming, Surrey, UK) with a 30 kg load cell operated at 1 mm/sec, cutting perpendicular to the muscle fibers [24]. The shear force for each sample was recorded and averaged, and the results were expressed as a load in Newtons (N).

### Analysis of data

Genotype frequencies in the MC4R gene were estimated by direct counting. Allele frequencies, observed (Ho) and expected (He) heterozygosity, fixation index (F), and Hardy–Weinberg equilibrium test (HWE) were estimated using the GenAlEx 6.5 software [25].

The variables did not present significant deviations from normality according to the Shapiro–Wilks test. Also, a descriptive analysis of the growth variables (BW, AWW, AW180, ASW, preWDG, and postWDG) and meat quality [pH, Warner-Bratzler shear force (WBSF), and Cook-loss (CL)] was carried out. Using a generalized linear model model (Y_ij_ = μ +G_i_ + ε_ij_) that included the fixed effect of genotype (G_i_, effect of the *i*th genotype AA, AG, and GG), the association between genotypes in the MC4R gene and growth and meat quality traits was determined. The results were expressed as the mean of the least squares and their standard errors.

The effect of the alleles of the MC4R gene on the growth traits and meat quality evaluated was determined by means of an allelic substitution analysis. For this, the genotype was transformed into quantitative information by assigning values of 0 to the GG genotype, 1 to the heterozygous genotype (AG), and 2 to the AA genotype, using a simple linear regression model *Y* = *β*_0_ + *β*_1_*X_i_*, where: Y is the dependent variable (BW, AWW, AW180, ASW, preWDG, postWDG, pH, WBSF, and CL), *β*_0_ is the intercept, *β*_1_ is the estimated regression coefficient for allelic substitution of the studied single nucleotide polymorphism (SNP), and *X_i_* is the number of alleles of the genetic variant (0, 1 or 2) [26]. Finally, Pearson correlations were performed between all the variables. All the statistical analyses of this research were carried out with the Jamovi software (version 2.3) [27].

CL was evaluated at 48 h postmortem. The external fat was removed from each sample, and they were lightly dried with absorbent paper and weighed (iw). The samples were cooked in plastic bags submerged in a water bath at 75°C until reaching an internal temperature of 70°C, which was maintained for 20 min [22]. During cooking, the internal temperature of the samples was monitored using the digital thermometer described above. Once cooked, the samples were cooled to room temperature, lightly dried with absorbent paper, and weighed again (fw). Afterward, the cooking loss was calculated as (iw/fw)*100 [21,22].

## Results

The heterozygous genotype was the most frequent, followed by the GG genotype (Table 1). The above made the frequency of the G allele of the MC4R gene higher (0.607 ± 0.001). The observed (Ho) and expected (He) heterozygosity values were similar, with a positive but insignificant *F* value. Thus, this particular polymorphism did not present significant deviations from the expected HWE theorists (Table 1).

The average BW in the lambs was 2.36 ± 0.63 kg, and the AWW was 12.75 ± 3.94 kg. During this period, the growth rate was 115.41 ± 42.84 gm/day. The AW180 was 31.21 ± 8.78 kg, and the growth rate in this period of time was 96.16 ± 29.19 gm/day (Table 2). In the meat quality traits in LTL muscle, the averages were 49.46 ± 17.64 N, 37.02% ± 13.29%, and 5.75 ± 0.12 units for WBSF, CL, and pH, respectively.

In growth traits, just BW was significantly associated with the polymorphism studied in the MC4R gene, with the best performance for the GG genotype (Table 3). In the same genotype, we found the highest AW180, with no differences between genotypes. On the other hand, the AA genotype showed the highest values for the AWW, ASW, preWDG, and postWDG variables.

None of the genotypes was significantly associated with the quality traits of the meat evaluated in the LTL muscle from OPC (Table 3). However, in AA-genotype animals, cooking losses tended to be lower (*p* = 0.09). While the cutting force (WBSF) was higher for this same genotype (*p* = 0.294).

**Table 1.** Allelic, genotypic frequencies and genetic diversity indices of the MC4R gene in OPC.

Genotypic frequencies			Allelic frequencies		Genetic diversity indices			
AA (*n =* 26)	AG (*n =* 80)	GG (*n =* 62)	A	G	Ho	He	F	HWE (*p*-value)
0.155	0.476	0.369	0.393 ± 0.001	0.607 ± 0.001	0.476	0.477	0.002	0.982

Ho: observed heterozygosity, He: expected heterozygosity, F: fixation index, HWE: Hardy–Weinberg equilibrium deviations.

**Table 2.** Averages and measures of dispersion for growth traitsand meat quality, measured in the LTL muscle in OPC sheep.

Traits		Mean	SE	Min	Max
Growth	BW (kg)	2.36	0.627	1.00	3.50
	AWW (kg)	12.75	3.935	6.04	25.54
	AW180 (kg)	19.67	5.291	9.21	33.33
	ASW (kg)	31.21	8.780	13.41	54.21
	Pre-WDG (gm)	115.41	42.843	42.56	246.04
	Post-WDG (gm)	96.16	29.198	35.05	174.04
Meat quality	WBSF (N)	49.46	17.636	15.20	94.54
	CL (%)	37.02	13.285	13.61	133.31
	pH	5.75	0.124	5.37	6.18

BW: birth weight, AWW: adjusted weaning weight at 90 days, AW180: adjusted weight at 180 days, ASW: adjusted slaughter weight, Pre-WDG: pre-weaning daily gain Post-WDG: post-weaning daily gain, WBSF: Warner-Bratzler shear force, CL: cook-loss.

**Table 3.** Effect of the G/A polymorphism on growth traitsand meat quality, measured in the LTL muscle in OPC sheep.

Traits		MC4R gene genotypes			*p*-value
		AA (*n =* 26)	AG (*n =* 80)	GG (*n =* 62)	
Growth	BW (kg)	1.95 ± 0.54^b^	2.24 ± 0.57^b^	2.69 ± 0.58^a^	<0.001
	AWW (kg)	12.84 ± 3.83	12.79 ± 4.05	12.65 ± 3.89	0.970
	AW180 (kg)	19.82 ± 0.50	19.36 ± 5.18	20.00 ± 5.59	0.768
	ASW (kg)	31.73 ± 8.26	30.78 ± 8.53	31.54 ± 9.40	0.830
	Pre-WDG (gm)	121.02 ± 41.67	117.25 ± 43.13	110.69 ± 43.18	0.514
	Post-WDG (gm)	99.29 ± 26.79	95.13 ± 28.01	96.19 ± 31.83	0.821
Meat quality	WBSF (N)	52.77 ± 17.29	50.38 ± 18.46	46.88 ± 16.59	0.294
	CL (%)	33.65 ± 9.19	38.95 ± 15.67	35.95 ± 10.93	0.090
	pH	5.76 ± 0.10	5.76 ± 0.14	5.75 ± 0.12	0.913

^a,b^ indicate statistical differences between genotypes.

BW: birth weight, AWW: adjusted weaning weight at 90 days, AW180: adjusted weight at 180 days, ASW: adjusted slaughter weight, pre-WDG: pre-weaning daily gain post-WDG: post-weaning daily gain, WBSF: Warner-Bratzler shear force, CL: cook-loss.

On the other hand, the BW varied significantly due to the effect of replacing the G allele with the A allele, decreasing it by 388 gm (Table 4). This effect was not significant for the other growth traits. However, the substitution effect showed a trend (*p* < 0.1) for the WBSF and CL variables, indicating an increase in the cutting force (0.313 N) and a decrease in cooking losses (−0.267%) when changing the G allele for A. In addition, the BW was only significantly correlated with the AWW at a low magnitude (Table 5). The strongest significant correlations were found between preWDG and AWW, as well as between postWDG and AW180 and ASW. Among the meat quality variables, the correlations were of medium magnitude, significant (*p* < 0.01), and had a great effect on pH. Finally, preWDG (*p* < 0.05) and AWW (*p* < 0.001) presented the highest correlations with the meat quality variables in LTL muscle from OPC.

**Table 4.** Effect of allelic substitution (1016G>A) on growth traitsand meat quality, measured in LTL muscle in OPC sheep.

Traits		β_0_	β_1_	*p*-value
Growth	BW (kg)	2.665	−0.388	0.001
	AWW (kg)	12.666	0.103	0.815
	AW180 (kg)	19.834	−0.209	0.725
	ASW (kg)	31.279	−0.089	0.928
	Pre-WDG (gm)	11.120	5.46	0.255
	Post-WDG (gm)	95.380	0.995	0.761
Meat quality	WBSF (N)	4.798	0.313	0.085
	CL (%)	37.233	−0.267	0.060
	pH	5.750	0.004	0.753

β0: model intercept, β1: allelic substitution effect of the SNP studied, BW: birth weight, AWW: adjusted weaning weight at 90 days, AW180: adjusted weight at 180 days, ASW: adjusted slaughter weight, Pre-WDG: pre-weaning daily gain Post-WDG: post-weaning daily gain, WBSF: Warner-Bratzler shear force, CL: cook-loss.

**Table 5.** Pearson correlations between growth traitsand meat quality in LTL muscle in OPC sheep.

Traits	BW (kg)	AWW (kg)	AW180 (kg)	ASW (kg)	Pre-WDG (gm)	Post-WDG (gm)	WBSF (N)	CL (%)
BW (kg)	----							
AWW (kg)	0.2*	----						
AW180 (kg)	0.12	0.63***	----					
ASW (kg)	0.07	0.62***	0.99***	----				
Pre-WDG (gm)	0.05	0.99***	0.62*	0.62*	----			
Post-WDG (gm)	0.0	0.61**	0.99***	0.99*	0.62*	----		
WBSF (N)	−0.03	0.26***	0.22**	0.22*	0.27*	0.23*	----	
CL (%)	0.02	0.28***	0.21**	0.21*	0.29*	0.21*	0.23**	----
pH	0.1	0.04***	0.06*	0.05*	0.03*	0.05	0.28***	0.26***

*p < 0.05, ** *p* < 0.01, ***p < 0.001.

BW: birth weight, AWW: adjusted weaning weight at 90 days, AW180: adjusted weight at 180 days, ASW: adjusted slaughter weight, preWDG: preweaning daily gain postWDG: postweaning daily gain, WBSF: Warner-Bratzler shear force, CL: cook-loss.

## Discussion

The MC4R gene has been associated with productive traits in cattle [28], pigs [29], chickens [30], goats [31], and sheep [13,19], making it a good candidate gene to be assessed in the OPC sheep. The evaluated transition (1016G > A) is located in the 3�-untranslated region (3�-UTR) of the MC4R gene. This region has regulatory functions, as it is involved in polyadenylation processes, nuclear transport, subcellular targeting, translation, and mRNA degradation [32]. Therefore, polymorphisms in its sequence may imply changes in gene expression, a principle demonstrated in various publications, not only for growth traits [32] but also in response to diseases [33].

Using PCR-restriction fragment length polymorphism with the *AciI* enzyme in the Karachai sheep breed, the allele frequencies were 0.65 and 0.35 for alleles A and G, respectively. While the frequencies of the genotypes were 0.47 for the AA homozygote, 0.37 for the heterozygote, and 0.16 for the GG homozygote [13]. In addition, in Merino sheep, by sequencing only the GA and GG genotypes, frequencies of 0.29 and 0.71, respectively, were found [19], which translated into a higher frequency of the G allele (0.86), similar to the results of this study. On the other hand, in Hu sheep [14], the frequency of the G allele was the lowest (0.23) and that of the AA genotype the highest (0.54). Differences in genotypic frequencies can be the product of mutation, genetic drift, migration, natural selection, and directed selection on production traits, which can increase the genetic differentiation of populations [13,19]. The observed (0.372) and expected (0.455) heterozygosity values reported by Gorlov et al. [13] in the Karachai breed, in the Merino breed (Ho: 0.29, He: 0.24) [19], and in the Hu breed (Ho: 0.38, He: 0.39) [14] were lower than those found in the OPC (Table 1), which suggests high genetic diversity in the OPC, reaching a value of 47.7%. The low differences between the observed and expected heterozygosity values in the OPC resulted in a low positive fixation index. As reported by Gorlov et al. [13] in the Karachai breed, by Zuo et al. [19] in the Merino breed, and by Song et al. [14] in the Hu breed, the *locus* evaluated in this investigation did not present significant deviations from the HWE.

Several studies have reported the behavior of growth variables in OPC lambs. The BW was lower than that presented by Montes-Vergara et al. [3] (2.8 ± 0.21 kg), Vergara et al. [4] (2.6 ± 0.6 kg), Palacios et al. [2] (2.8 ± 0.8 kg), Noriega et al. [5] (3.2 ± 0.5 kg), and Lenis et al. [12] (3.10 ± 0.70 kg). These differences are associated with the number of lambs at birth [34], ewe age [35,36], the feeding management of the pregnant ewes during the last third of gestation [37], and the male used during the breeding season [3].

The weaning weight presented here is higher than that reported in other studies: 11.6 ± 3.6 kg [3], 11.2 ± 3.7 kg [4], and 10.3 ±1.7 kg [5]. But less than the weight found by Palacios et al. [2] (15.1 ± 3.9) and Lenis et al. [12] (18.5 ± 0.7). Some research investigations suggest that in addition to lactation duration and the ewe’s ability to provide maternal care to its lamb [38], the lamb’s sex [2,4], the ewe’s parturition number [35,36], the climatic season during which lactation takes place [3], and the male [3], are determinants of lamb performance at this age. Table 5 shows a low correlation (0.20) between BW and AWW (*p* < 0.05). Works performed in Sardi [39] and Merino [40] sheep breeds for this correlation present values of 0.68 and 0.27, respectively. This confirms that the maternal prenatal effect can favorably affect the growth performance of the OPC lamb.

The preWDG value is higher than that reported for this breed by Montes-Vergara et al. [3] (95 ± 40 gm) and Vergara et al. [4] (95 ± 40 gm), but lower than that reported by Palacios et al. [2] (137.3 ± 43.3 gm) and Lenis et al. [12] (128.3 ± 28.3 gm). Certainly, the ewe’s colostrum and milk production capacity [36], the feed supply to the ewe and their lamb [2,4], the number of lactating lambs [36], the body condition [5], and the health status of the ewe and its lamb [5] may be associated with these variations. Likewise, preWDG, as expected, was positively and significantly correlated with postweaning weights.

On the other hand, the postWDG found here is higher than other works already cited in the OPC [4,2,12]. The postWDG was positively and significantly correlated with the weights measured after weaning (Table 5), which were lower than those presented in other studies for the weights at [5] and ASW [2], which could be due to the effect on animal management practices related to feeding and health-care management during lamb fattening [7].

There are no reports on the measurement of WBSF and CL in OPC lambs, so our results are the first report on the subject. WBSF values in the LTL muscle of 23.31 N are presented in Merino [41], 31.4 N in Dorper x Katahdin [42], 24.38 N in Santa Ines x Dorper [43], and 45.7 N in Norduz lambs [44]. Destefanis et al. [45] classified meat into five groups according to its tenderness: very tough (WBSF >62.59 N), tough (52.78 N < WBSF < 62.59 N), acceptably tender (42.87 N < WBSF < 52.68 N), tender (32.96 N < WBSF < 42.77 N), and very tender (WBSF < 32.96 N), classifying the OPC meat as acceptably tender. Other sources of intrinsic variation besides breed and its genes in meat tenderness include sex, age, slaughter weight, and muscle, where WBSF is measured [46]. In this regard, the evaluated polymorphism did not affect this characteristic. However, the WBSF showed a positive correlation (*p* < 0.05) with weight at different ages and with weight gain, as found by Carrillo-Muro et al. [42]. On the other hand, carcass pH, storage temperature, maturation time, marbling, and cooking procedure are classified as postmortem factors that modify WBSF [46].

The CL found in the LTL of OPC lambs is higher than those reported for this same muscle in Dorper x Katahdin 24.8% [42], Santa Ines x Dorper 23.65% [43], and Norduz 30.51% [44] lambs. In addition to this racial factor and the variations in genes of interest between them, it is understood that the pH of the meat is the most important factor on which the LC depends. However, sex, the amount of fat in the muscle, and the time of maturation can also affect it [46]. The latter is an effect of proteolytic changes caused principally by calpains, which cause the degradation of muscle tissue proteins and induce changes in the micro and ultrastructure of muscle fibers, facilitating cooking losses [46]. The analyses showed positive correlations (*p* < 0.05) between LC and weight at different ages. However, some authors have found similar findings [47], justified by the fact that sheep meat takes longer to reach the degree of doneness, which means a higher CL. Others, conversely, assure that age instead of weight is more decisive [46].

In 24 OPC carcasses fed with different levels of cottonseed inclusion, meat pH at 24 h postmortem was lower than that found here and varied between 5.51 and 5.55 measured in the *longissimus dorsi* muscle [8], suggesting that these animals were not subjected to stress during slaughter. A similar pH value (5.75 ± 0.25 units) to that of this work, determined in the *longissimus dorsi* muscle of 10-month-old OPC lambs (*n* = 36), is reported by Trujillo et al. [48]. On the other hand, Albarracín et al. [6] found that in 60 F1 OPC x Dorper lambs, which had fasted for 18 h, the pH at 24 h postmortem measured on the shoulder of the carcass varied between 5.9 and 6.1, a range similar to the value found in the present work. Finally, in 883 carcasses of undetermined breeds in a slaughterhouse in Colombia, at 24 h postmortem, in the *Biceps femoris* muscle, the pH recorded was higher (5.85) compared to this work, but with differences between males (5.84 ± 0.01 units) and females (5.94 ± 0.03 units) [7].

The pH value was 5.75 ± 0.124 units, which can be considered high. The above suggests that the lambs arrived at the slaughter plant with low glycogen reserves in their muscles [7]. Among the antemortem factors that can cause this situation are the production system (feeding, health, animal management, and climatic conditions), the breed and genetic condition of the individual itself, and transport [9].

Our work showed a medium-sized but positive and significant correlation (*p* < 0.001) between pH and WBSF and CL traits, suggesting a decrease in tenderness and an increase in cooking losses with increasing pH. Contrary results are shown by Wyrwisz et al. [49] for the correlation between pH and WBSF (*r* = −0.65; *p* ≤ 0.01), but similar for pH and CL (*r* = 0.74; *p* ≤ 0.01). This difference is associated with the maturation time of the meat, since in the research by Wyrwisz et al. [49], the correlation was determined after 21 days of maturation; thus, the increase in pH recorded during this stage should have favored the release of proteolytic products that could decrease WBSF (improving tenderness) by denaturing the protein structure with the consequent increased cooking losses.

Regarding the association of the 1016G > A variant in the MC4R gene with growth traits and meat quality, in the Karachai breed, the BW was higher for the AG genotype without statistical differences between genotypes [13]. While slaughter weight and estimated weight gain were higher for the AA genotype (*p* < 0.05). In the Merino breed, this mutation did not affect BW, but it did affect weight at 120 and 180 days and daily weight gain at these ages (*p* < 0.05), with a better value for heterozygous animals; likewise, these animals also had higher back fat (cm) and loin-eye area [19]. On the other hand, the genotype under study in the Hu breed did not affect the BW but the AWW, with better weight in the GG animals [14]. Our data show a higher BW for the GG genotype (*p* < 0.05), without statistical differences for the other growth variables (*p* > 0.05), but a better value for the AA genotype.

In Karachai sheep, the effect of this SNP on carcass quality traits was evaluated [13]. Their results show that sheep with the AA genotype have heavier hot and cold carcasses as well as higher fat content. intramuscular and in the tail, with respect to the other genotypes.

It had been proposed that, in addition to variations within the gene, the low-fat content of the diet, which is low in tropical grasses, accentuates fat deposition [14]. Additionally, in combination with neuropeptide Y, which stimulates feed intake and increases growth hormone release in sheep, muscle deposition [14] is favored over fat deposition, suggesting a differential growth pattern between ruminant and nonruminant mammals related to your eating habits.

The mean effect of allelic substitution showed a significant decrease in BW in the mutant allele (A). This substitution did not affect the other growth variables and showed a slight upward trend in WBSF. Song et al. [14] refer to this allele as the beneficial one since it improves weight, muscle growth, and back fat deposition.

Finally, some recommendations derived from the results of this work include enhancing the sample size by including other OPC populations, evaluating other SNPs reported in the MC4R gene, and identifying more candidate genes associated with growth and meat quality. Nongenetic factors can affect the variables studied. Thus, it is necessary to include those factors in new research. In addition, for future research, it is considering subjecting the meat to different maturation times and performing measurements in other muscles of palatable and economic interest. In this sense, we considered recommending conducting genome-wide association work to identify and validate new regions associated with quantitative trait locis.

## Conclusion

In this work, it was possible to find the 1016G > A polymorphism in the study breed, with a high frequency of the GG genotype and the G allele, high heterozygosity, and HWE. The pH found in the LTL muscle suggests stress in slaughter lambs with positive and significant correlations with texture and cooking losses, which translate into a decrease in meat tenderness. These results suggest that pH is an important indicator of meat quality. In general, OPC meat can be described as acceptably tender. However, future investigations should be carried out to evaluate the effect of maturation time on this characteristic. The genotype and allelic substitution effects affected the BW of the lambs but not the other growth traits or the meat quality of the LTL muscle. The great genetic variation found in this gene suggests the need to genotype other variations reported in the MC4R gene and in other candidate genes; likewise, to expand this study to other OPC populations where the distribution of the genotypes is possibly different, which would help support these results. The identification of animals carrying the allelic variants of interest will contribute to the successful implementation of a breeding program assisted by molecular markers.

## List of abbreviations

AA: AA genotype, AG: AG genotype, ASW: adjusted slaughter weight, AW180: adjusted weight at 180 days, AWW: adjusted weaning weight at 90 days, BW: birth weight, CL: cook-loss, GG: GG genotype, LTL: *longissimus thoracis et lumborum* muscle, MC4R: melanocortin-4 receptor, OPC: Colombian hair sheep, PCR: polymerase chain reaction, PostWDG: postweaning daily gain, PreWDG: preweaning daily gain, SNP: single nucleotide polymorphism, WBSF: Warner-Bratzler shear force.
